# Neuroprotection by acrolein sequestration through exogenously applied scavengers and endogenous enzymatic enabling strategies in mouse EAE model

**DOI:** 10.1038/s41598-024-56035-z

**Published:** 2024-03-12

**Authors:** Jonathan Tang, Anna Alford, Gary Leung, Melissa Tully, Riyi Shi

**Affiliations:** 1 Department of Basic Medical Sciences, College of Veterinary Medicine, Purdue University, West Lafayette, IN 47907 USA; 2 Weldon School of Biomedical Engineering, Purdue University, West Lafayette, IN 47907 USA; 3 Center for Paralysis Research, Purdue University, West Lafayette, IN 47907 USA; 4 MSTP Program, Indiana University School of Medicine, Indianapolis, IN USA

**Keywords:** Biochemistry, Neuroscience

## Abstract

We have previously shown that the pro-oxidative aldehyde acrolein is a critical factor in MS pathology. In this study, we found that the acrolein scavenger hydralazine (HZ), when applied from the day of induction, can suppress acrolein and alleviate motor and sensory deficits in a mouse experimental autoimmune encephalomyelitis (EAE) model. Furthermore, we also demonstrated that HZ can alleviate motor deficits when applied after the emergence of MS symptoms, making potential anti-acrolein treatment a more clinically relevant strategy. In addition, HZ can reduce both acrolein and MPO, suggesting a connection between acrolein and inflammation. We also found that in addition to HZ, phenelzine (PZ), a structurally distinct acrolein scavenger, can mitigate motor deficits in EAE when applied from the day of induction. This suggests that the likely chief factor of neuroprotection offered by these two structurally distinct acrolein scavengers in EAE is their common feature of acrolein neutralization. Finally, up-and-down regulation of the function of aldehyde dehydrogenase 2 (ALDH2) in EAE mice using either a pharmacological or genetic strategy led to correspondent motor and sensory changes. This data indicates a potential key role of ALDH2 in influencing acrolein levels, oxidative stress, inflammation, and behavior in EAE. These findings further consolidate the critical role of aldehydes in the pathology of EAE and its mechanisms of regulation. This is expected to reinforce and expand the possible therapeutic targets of anti-aldehyde treatment to achieve neuroprotection through both endogenous and exogenous manners.

## Introduction

Multiple sclerosis (MS) is an inflammatory, demyelinating disease of the brain and spinal cord which affects approximately 2.5 million individuals worldwide, often striking young adults without warning^1–4^. Patients experience a variety of motor, sensory, and cognitive deficiencies^1,3,5^. Unfortunately, due to the incomplete understanding of the etiology of MS, current available therapies remain largely unsatisfactory, as they mainly focus on immunomodulation or symptomatic relief with limited success^2^. As such, the long-term trajectory and prognosis for most MS patients, a continual worsening of symptoms until death, is not significantly altered by the current standards of care^6–11^. Thus, a major priority in this field is to identify key pathological factors that can provide new and effective therapeutical targets to deter the pathological progression of MS.

There is strong evidence that reactive oxygen species are the mediators of MS pathology^12–16^. However, antioxidant therapeutic strategies alone have shown limited success in mitigating disease progression^14,17,18^. Recently, emerging scientific findings suggest that reactive aldehydes, products of free radical-instigated lipid peroxidation, may play a critical role in the pathogenesis of MS^19–23^. Acrolein, a toxic reactive aldehyde, is both a product and catalyst of oxidative stress and inflammation^24,25^. As such, it can induce a vicious cycle wherein it begets and worsens the mechanisms by which it is created, thereby amplifying its effects^24–26^. Therefore, acrolein may provide a better target to suppress oxidative stress in MS.

The involvement of acrolein in neuroinflammation and potentially MS has been demonstrated in experimental autoimmune encephalomyelitis (EAE) models^19,20,22,23^. Acrolein levels are significantly elevated in EAE mice in a manner which temporally correlates with the development of behavioral deficits^19,22^. Previous studies using various preparations from cell culture to animal models indicate that acrolein, at micromolar concentrations, can cause both demyelination^27^ and axonal degeneration^28^, two hallmarks of MS pathology^4,29,30^. The detrimental effects of acrolein are mediated by its ability to attack protein, lipids, and DNA, thereby perpetuating oxidative stress^28,31–33^, disrupting myelin and membrane integrity^27,28,33,34^, and inhibiting key mitochondrial enzymes^2,25,32,35,36^, pathologies all implicated in MS^2,36^. To further support the role of acrolein in clinical MS, recent studies have demonstrated an elevation of acrolein among MS patients compared to healthy control individuals^22,37^.

Due to the strong evidence implicating the pathological role of acrolein in EAE, we also initiated efforts to achieve neuroprotective effects through the reduction of acrolein. Specifically, the acrolein scavenger hydralazine has been shown to effectively sequester acrolein in EAE, reducing both motor deficits and myelin destruction^19,22^. This offers direct evidence supporting acrolein as an effective therapeutic target for neuroprotection in EAE. In addition, a recent study reported that hydralazine was able to reduce acrolein in a human MS patient, further underscoring acrolein as a target for pharmaceutical intervention^37^. In order to demonstrate that the acrolein-scavenging capability among acrolein scavengers is the main reason for their neuroprotection in EAE, it is necessary to test other structurally distinct acrolein scavengers. In addition, to assess the possibility of adapting anti-acrolein therapy for MS, it is important to test the extent of its neuroprotective effects by initiating treatment after the emergence of motor deficits. Furthermore, even though acrolein scavengers have been shown to mitigate MS-related motor deficits, they have not been tested to alleviate the accompanying neuropathic pain in EAE. This is a form of neurological dysfunction that is receiving increasing attention among MS patients and should therefore be included in the analysis of functional neuroprotection in EAE^38–40^.

It is increasingly clear that acrolein can be produced or metabolized by a variety of endogenous enzymes^35,41^. The correlation of these proteins and the dynamics of acrolein may provide insights into the mechanisms of acrolein surge and inflammation, suggesting measures for reducing acrolein and related inflammation. Myeloperoxidase (MPO), a pro-inflammatory enzyme and key player in inflammatory flares in MS^42,43^, is also known to produce acrolein^35,41^. Additionally, acrolein is known to induce inflammation^44,45^. Therefore, acrolein may likewise stimulate the production of MPO, forming a vicious cycle that synergistically drives the spread of inflammation when acrolein is elevated. On the other hand, aldehyde dehydrogenase 2 (ALDH2), a mitochondrial enzyme, is known to scavenge toxic aldehydes such as acrolein^46–50^. However, few studies have explored the role of ALDH2 in detoxifying acrolein in EAE, and none have taken advantage of using ALDH2-deficient transgenic mice^46,51^. Therefore, elucidating the correlations of these key factors with acrolein may not only help to deepen our understanding of MS pathology, but may perhaps shed light on novel treatment options.

In summary, the current study was designed to further solidify the notion that anti-acrolein therapies using exogenously-applied scavengers are effective neuroprotective measures that can not only mitigate MS-like motor deficits, but also sensory hyperreflexia. In addition, this study aimed to explore the role of ALDH2 in acrolein clearance as well as the mitigation of related neuronal dysfunctions through this endogenous mechanism. Using a mouse EAE model, we have demonstrated that acrolein scavengers can offer relief of motor symptoms not only when administered on the day of induction, but also when they are delayed until after motor deficits emerge. In addition to the alleviation of motor symptoms, hydralazine can also mitigate hyperreflexia in EAE mice when applied immediately after induction. Besides hydralazine, phenelzine, another acrolein scavenger with a distinct chemical structure, is also capable of alleviating motor symptoms, further strengthening the conclusion that these beneficial effects are the result of acrolein scavenging. Furthermore, up or down regulation of acrolein is accompanied by similar changes in MPO levels, suggesting an anti-inflammatory effect of acrolein scavenging and a close interaction of these two pathological factors in EAE. In addition, we have shown that the mitochondrial enzyme ALDH2 plays a critical role in suppressing acrolein in EAE mice. The suppression of ALDH2 through genetic manipulation leads to clear elevations in acrolein and MPO, and more importantly, worsening motor and sensory dysfunctions. By contrast, Alda-1, a potent ALDH2 stimulator, could lower acrolein, alleviating motor and sensory dysfunction. Taken together, this data not only further validates the pathological role of acrolein in EAE, but also strengthens the notion that anti-acrolein therapy is a novel strategy, either through exogenous or endogenous mechanisms, that can offer significant neuroprotection in MS pathologies.

## Methods

### Animals

Female C57BL/6 mice (8 weeks old) were purchased from Envigo (Indianapolis, IN) and allowed to acclimate to laboratory housing facilities for a minimum of one week prior to experimentation. All the animals were randomly assigned into various experimental groups. Behavioral assessments were conducted by someone with no knowledge of the identity of the mice. All methods were carried out in accordance with relevant guidelines and regulations. Specifically, the animal procedures were performed under protocols approved by the Purdue Animal Care and Use Committee. The methods are reported in accordance with ARRIVE guidelines.

### Induction of EAE and motor function scoring

Mice between 9 and 12 weeks of age were injected subcutaneously with 0.1 mL MOG_35–55_/CFA emulsion (EK-2110, Hooke Laboratories) over rostral and caudal ends of the spinal column. 0.1 mL of deconjugated pertussis toxin (1 µg/mL) was administered intraperitoneally 60–90 min following injection of the emulsion, and again between 22 and 24 h later. Motor function was scored daily using an established 5-point system to assess the degree of paralysis. Scores were assigned in the following manner: 0-No deficit, 1-Tail paresis, 2-Hind limb paresis or ataxia without leg dragging, 3-Hind limb paresis with one or both hind limbs dragging, 4-Complete hind limb paralysis, 5-Moribund. Humane endpoints requiring euthanasia prior to the experimental timepoints included loss of over 20% body weight or attaining a motor function score of 5. Animals euthanized due to reaching a humane endpoint were assigned a motor score of 5 for the remaining duration of the experiment.

### Acrolein scavenger treatments

All drugs were dissolved in phosphate-buffered saline and sterilized with a 0.45 μM filter prior to storage at 4 C. Hydralazine hydrochloride (Millipore Sigma, St. Louis, MO) was administered at a dose of 1 mg/kg daily via intraperitoneal (IP) injection starting from either the day of induction or upon reaching a motor score of 1 and continuing through the duration of the study. Phenelzine (Millipore Sigma) was administered at 15 mg/kg daily via IP injection starting from the day of induction to further elucidate the role of acrolein scavenging in alleviating EAE-related motor deficits. Experimental groups not receiving pharmaceutical intervention received 0.1 mL phosphate-buffered saline. The doses of hydralazine and phenelzine used in the current study are known to be safe and effective in trapping acrolein in rodents based on prior studies^19,52,53^.

### Alda-1 treatment

Alda-1 (Cayman Chemical, Ann Arbor, MI) was dissolved in a vehicle consisting of 50% PEG-400 in DMSO. The Alda-1 treatment started immediately after the induction. Treatment animals were given 50 mg/kg by intraperitoneal injection daily, while animals in other groups received an equivalent volume of the vehicle.

### Assessment of mechanical hyperreflexia

Mechanical hyperreflexia was evaluated using calibrated von Frey filaments to quantify paw withdrawal thresholds in response to a known mechanical stimulus. Animals were placed on an elevated metal mesh platform, covered by a transparent plastic container, and allowed to acclimate for a minimum of 10 min prior to assessment. Calibrated von Frey filaments were applied to the plantar aspect of the hind paw with adequate bending force for between 3 and 5 s. As with our previous studies, the up-down method was utilized to determine 50% withdrawal thresholds with brisk movement of the affected limb being defined as a positive reaction^54–56^. The average result of the two limbs was recorded for each animal. These assessments took place prior to induction as well as on day 10 post induction to reduce false negatives resulting from hind limb motor inhibition.

### TRPA1 expression determination

Lumbar dorsal root ganglia were collected on day 10 post induction for healthy control, EAE, and EAE mice treated with hydralazine. Tissue was homogenized in Trizol and RNA was isolated via chloroform extraction followed by isopropanol precipitation. Isolated mRNA was quantified using a Nanodrop 2000c (Thermo Scientific). cDNA was synthesized using an iScript cDNA synthesis kit (Bio-Rad) according to the manufacturer’s instructions. Primers for Transient Receptor Potential A1 (TRPA1) were 5ʹ-TGCTGCAGAAAAAAATCAAGTTGA-3ʹ and 5ʹ-CCTTGGCTGAGAAGAAACTTTACACT-3ʹ. 18S rRNA was used as a reference with the primers 5ʹ-GCAATTATTCCCCATGAACG-3ʹ and 5ʹ-GGCCTCACTAAACCATCCAA-3ʹ^55–57^. Quantitative PCR was conducted using the iQ SYBR Green Supermix (BIO-RAD) and a Lightcycler 480 II (Roche) according to the manufacturer’s specifications. A pooled mixture consisting of equal parts cDNA from each control animal was used as the reference sample for the 2^−ΔΔCT^ method.

### Transgenic mice with aldehyde dehydrogenase deficiency

The colony of transgenic (TG) mice (ALDH2*2), generated at Stanford University, was later established at Purdue University. The detailed characteristics of ALDH2*2 TG mice can be found in the relevant publications^46,58^. While developmentally normal, ALDH2*2 mice only have less than 10% of normal ALDH2 function, replicating the same phenomenon in humans^46,58^.

### Isolation of spinal cord

Animals were first given Ketamine (90 mg/kg) and Xylazine (10 mg/kg) via intraperitoneal injection for euthanasia prior to perfusions. Following the injection of Ketamine and Xylazine, animals were then perfused transcardially with an oxygenated Krebs solution. The spinal cord was harvested by extracting the spinal column and removing the surrounding bone followed by fixation in 4% paraformaldehyde for 24 h.

### Immunohistochemistry

Fixed spinal cords were cryoprotected by incubating for 48 h in a 30% sucrose solution at 4 C. 1 cm segments were cut from the thoracic spinal cord, embedded in PolyFreeze (Polysciences, Warrington, PA), and frozen in a slurry of dry ice and 2-propanol. 25 μM sections were cut using a cryostat microtome (Thermo Scientific, HM525 NX) and stored in 0.1% sodium azide in phosphate buffered saline. Prior to staining, sections were rinsed with phosphate buffered saline for 5 min to remove any remaining embedding compound followed by a 30-min incubation in 3% hydrogen to quench endogenous peroxidase activity. Sections were permeabilized in 3% Triton-X in phosphate buffered saline and blocked with 5% normal donkey serum in 0.3% Triton-X for two hours. After washing three times with PBS, sections were incubated in the primary antibody at 4 C for 24 h. This study utilized the following antibodies: mouse anti-acrolein antibody (1:1000, 10A10, SMC-505, StressMarq); mouse myeloperoxidase antibody (1:500, GTX54393, GeneTex). Sections were then washed four times with PBS and incubated in the secondary antibody for 2 h at room temperature. This was followed by washing the slides three times with 0.1% Triton-X in PBS prior to incubation in avidin–biotin complex according to the manufacturer’s instructions (Thermo Scientific, Catalog No 32020). After an additional three washes in 0.1% Triton-X, sections were developed with a Pierce DAB substrate kit (Thermo Scientific, Catalog No. 34002) for five minutes and rinsed in distilled water. Sections were dehydrated with increasing concentrations of ethanol prior to mounting on positively charged slides (Polysciences, Warrington, PA) and applying coverslips with Permount. DAB stain intensity was determined with ImageJ.

### Statistical analysis

All data are presented in the format of mean ± standard error of the mean (SEM). One way ANOVA with Tukey’s LSD or Student’s t-test was used for statistical assessment (α = 0.05).

## Results

### Acrolein scavengers hydralazine and phenelzine mitigate the severity and delay the onset of motor symptoms in EAE mice

**Figure 1:**
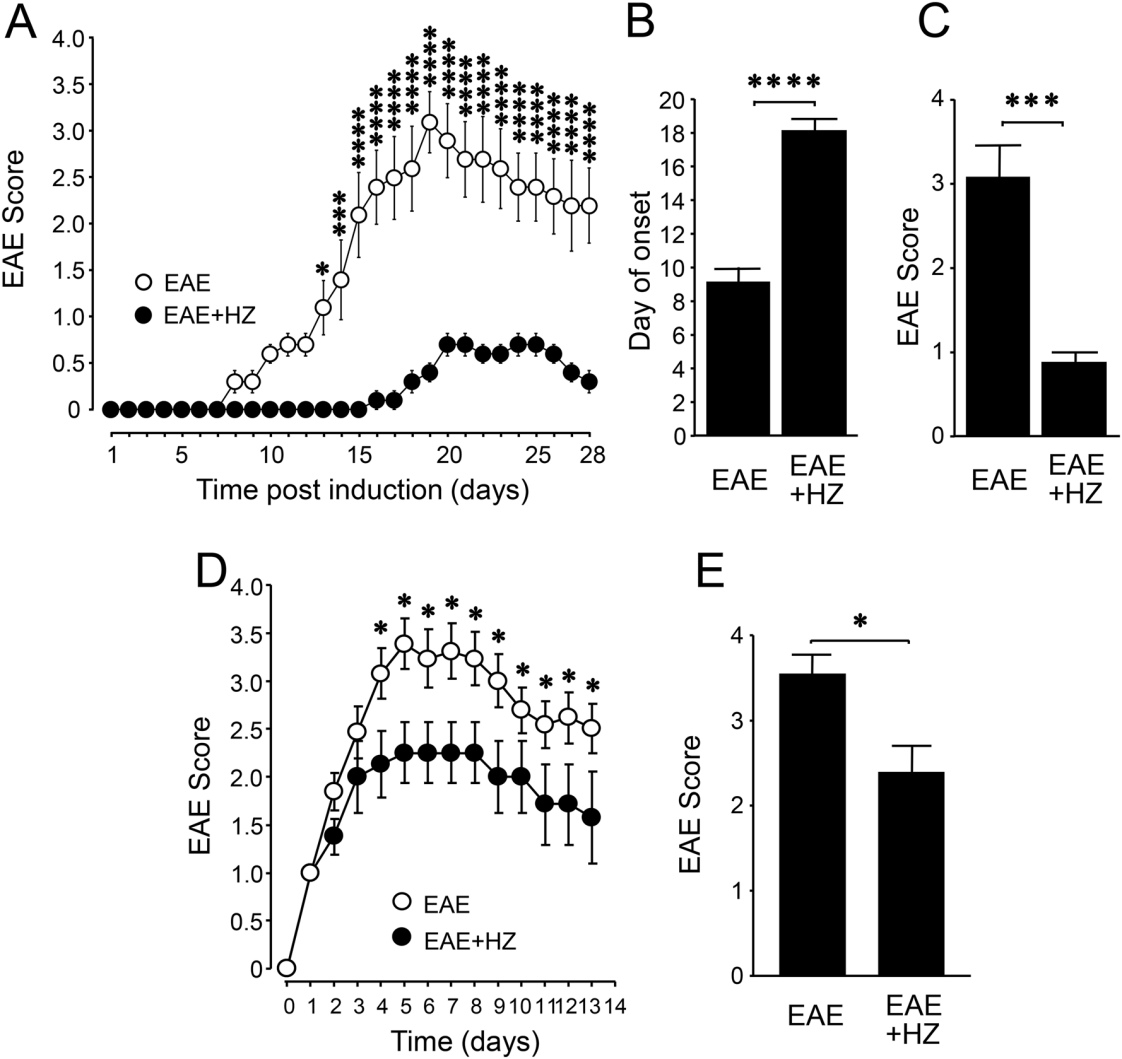
EAE motor symptom scores of EAE mice with and without hydralazine treatment commencing at the induction or symptom emergence. (**A**) Symptom scores over time for hydralazine and sham treatment starting at the induction in EAE mice. Hydralazine treatment significantly reduced the severity of symptoms starting on day 13, contributing to both later apparent onset and lower peak severity (comparison between EAE and EAE + HZ). (**B**) Hydralazine treatment delayed the average day of onset, defined as the day upon which the motor score reached 1.0, from 9.2 ± 0.73 days post induction to 18.2 ± 0.66 days post induction. (**C**) Hydralazine treatment reduced peak severity of motor impairment from 3.1 ± 0.37 to 0.9 ± 0.1. (**D**) Delaying hydralazine treatment until the appearance of motor symptoms significantly reduced disease severity starting on the fourth day of treatment and continuing until the end of the study (comparison between EAE and EAE + HZ). (**E**) Delayed treatment significantly reduced peak motor symptom scores from 3.54 ± to 0.24 to 2.38 ± 0.32. For A-E, *p < 0.05, **p < 0.01, ***p < 0.001, ****p < 0.0001.

There were two groups of mice in the experiment involving the treatment of hydralazine (HZ), a hydrazine-based acrolein scavenger. One group of EAE mice was treated with hydralazine (EAE + HZ) at a dose of 1 mg/kg daily starting from the day of induction (N = 5). In the sham-treated group (EAE), equal amounts of saline were injected daily (N = 5). Both hydralazine and saline treatments were carried out for 28 days post induction. For all the mice in both groups, motor symptoms were scored using an established 5-point scale and recorded for up to 28 days post induction (Fig. 1A). In the sham-treatment group, the mice displayed typical motor deficits characteristic of this animal model of EAE. However, hydralazine-treated animals exhibited an overall reduction of severity beginning at day 13, as well as a delay in the onset of motor deficits, when compared to the sham-treatment group (Fig. 1A). Specifically, hydralazine treatment delayed the onset of EAE symptoms, defined as the day post induction of reaching a score of 1.0, with the average day of onset shifting from 9.2 ± 0.37 days post induction in sham-treated mice to 18.2 ± 0.66 days post induction in phenelzine-treated mice (p < 0.0001) (Fig. 1B). Furthermore, the peak symptom severity was reduced from 3.1 ± 0.37 in sham-treated animals to 0.9 ± 0.1 in those receiving hydralazine (p < 0.001) (Fig. 1C). A peak severity (highest score) was recorded from each mouse, and then an average was generated among all the mice in the same group (EAE, or EAE + HZ). Delaying hydralazine treatment until the onset of motor symptoms also corresponded to a significant reduction in motor symptom severity. Specifically, animals treated with hydralazine (EAE + HZ, n = 8), at a daily dose of 1 mg/kg, showed significantly less severe motor scores starting on the fourth day of treatment when compared to sham-treated animals (EAE, n = 10) (p < 0.05) (Fig. 1D). Furthermore, peak symptom severity was attenuated from 3.54 ± 0.24 in sham-treated EAE mice to 2.38 ± 0.32 in hydralazine-treated mice (p < 0.05) (Fig. 1E). A peak severity (highest score) was recorded from each mouse, and then an average was generated among all the mice in the same group (EAE, or EAE + HZ).

**Figure 2:**
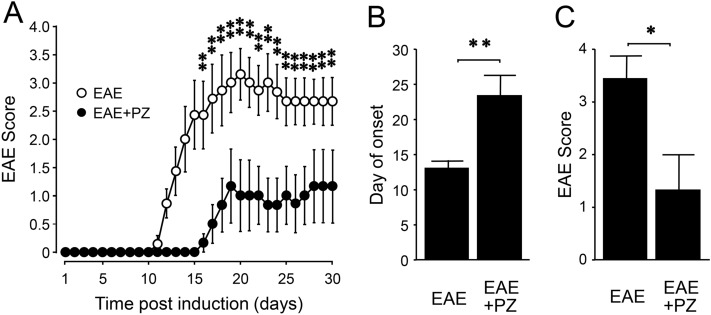
EAE motor symptom scores of EAE mice with and without phenelzine treatment commencing at the induction. (**A**) Symptom scores over time for phenelzine and sham treated EAE mice. Phenelzine treatment significantly reduced the severity of symptoms starting on day 16, contributing to both later apparent onset and lower peak severity (comparison between EAE and EAE + PZ). (**B**) Phenelzine treatment delayed the average day of onset, defined as the day upon which the motor score reached 1.0, from 13.14 ± 1.03 days post induction to 23.5 ± 2.92 days post induction. (**C**) Phenelzine treatment reduced peak severity of motor impairment from 3.43 ± 0.43 to 1.33 ± 0.67. For A-C, *p < 0.05, **p < 0.01.

To examine if phenelzine (PZ), also a hydrazine-based acrolein scavenger, can provide comparable relief in EAE motor symptoms, we carried out experiments similar to those conducted with HZ to assess its effect in EAE mice. There were two groups of mice in the experiment involving treatment with phenelzine. One group of EAE mice was treated with phenelzine (EAE + PZ) at a dose of 15 mg/kg daily starting from the day of induction (N = 6). In the sham-treated group (EAE), equal amounts of saline were injected daily (N = 7). Both phenelzine and saline treatments were carried out for 30 days post induction. Again, motor symptoms were scored using an established 5-point scale and recoded for up to 30 days post induction (Fig. 2A). In the sham-treatment group, the mice displayed typical motor deficits characteristic of this animal model of EAE. However, phenelzine-treated animals exhibited an overall reduction of symptom severity beginning at day 16, as well as a delay in the onset of motor deficits, when compared to the sham-treatment group (Fig. 2A). Specifically, phenelzine treatment delayed the onset of EAE symptoms, defined as the day post induction of reaching a score of 1.0, with the average day of onset shifting from 13.14 ± 1.03 days post induction in sham-treated mice to 23.5 ± 2.92 days post induction in the phenelzine-treated mice (p < 0.01) (Fig. 2B). In fact, half of the total animals in the phenelzine-treated group did not develop EAE symptoms, and thereby received a value of 30 as the day of symptom onset. Furthermore, the peak symptom severity was reduced from 3.43 ± 0.43 in sham-treated animals to 1.33 ± 0.67 in those receiving phenelzine (p < 0.05) (Fig. 2C).

### Hydralazine alleviates mechanical hyperreflexia in EAE mice

**Figure 3:**
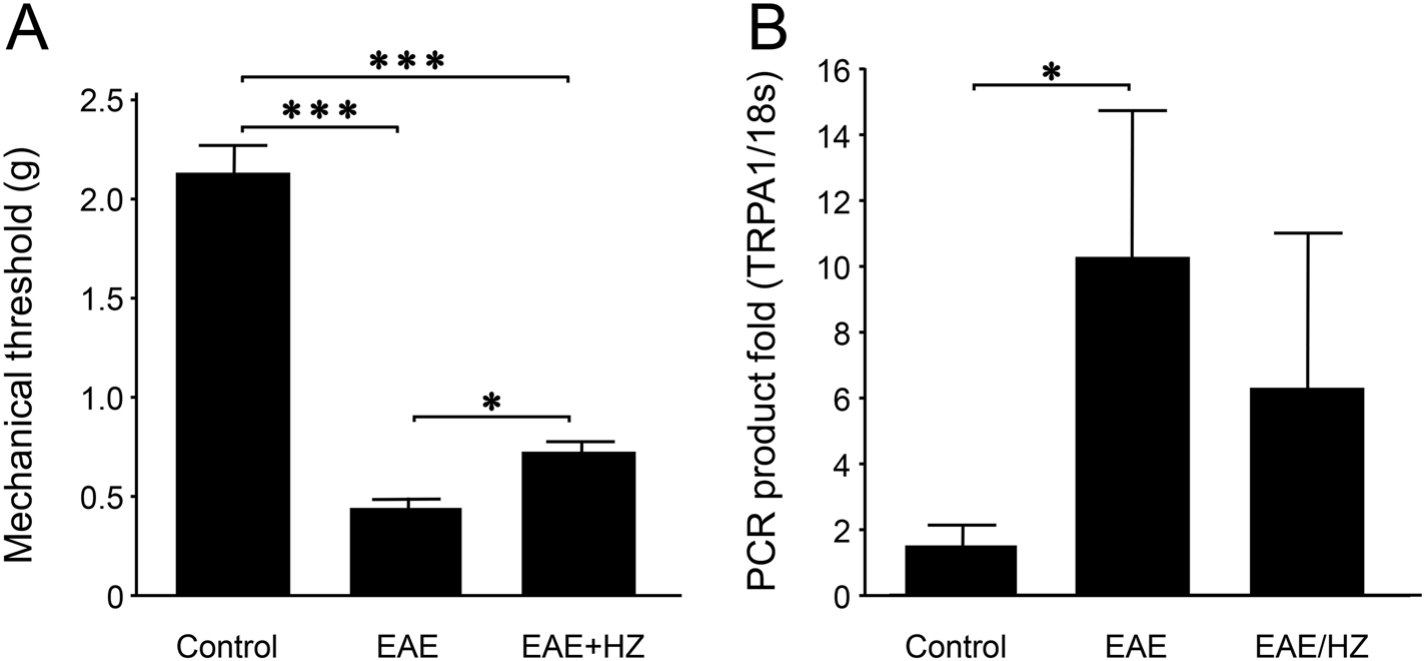
Acrolein scavengers alleviated hypersensitivity and demonstrated a tendency to mitigate elevated expression of TRPA1 on day 10 post induction in EAE mice. (**A**) Effect of hydralazine treatment (initiated at the induction) on mechanical hyperreflexia assessed on day 10 post induction. EAE mice exhibited significantly lower hind paw withdrawal thresholds at 0.45 ± 0.05 g compared to healthy controls, which showed an average withdrawal threshold of 2.11 ± 0.15. Hydralazine treatment partially alleviated this to 0.76 ± 0.09 g, which is improved compared to untreated EAE mice, but still exhibited higher sensitivity than the control group. (**B**) Relative expression of TRPA1 on day 10 post induction. TRPA1 expression in EAE mice was elevated compared to the control group, with a 10.25 ± 4.4 fold increase in expression compared to the control group (1.54 ± 0.58 fold). Hydralazine-treated (initiated at the induction) animals trended towards lower TRPA1 expression when compared to untreated EAE mice, but this difference was not statistically significant due to high variance in both groups. For (**A**, **B**), *p < 0.05, ***p < 0.001.

Calibrated von Frey filaments were used to assess sensory hypersensitivity^56^. When examined on day 10 post induction, EAE mice exhibited significantly lower mechanical response thresholds at 0.45 ± 0.05 g (n = 10) compared to healthy control animals at 2.11 ± 0.15 g (n = 5) (p < 0.001) (Fig. 3A). Hydralazine treatment, at 1 mg/kg daily starting from the day of induction (EAE + HZ, 0.76 ± 0.09, n = 10), significantly improved mechanical thresholds when compared to EAE mice (p < 0.05). However, the mechanical thresholds in the hydralazine-treated group remained significantly lower than the control group (p < 0.001) (Fig. 3A).

### Elevation of TRPA1 gene expression in the dorsal root ganglia of EAE mice

The mRNA gene expression of Transient Receptor Potential A1 (TRPA1), a pain-sensing channel that can be activated by acrolein, was measured in the dorsal root ganglia by quantitative polymerase chain reaction relative to 18S^55–57^. EAE mice exhibited a 10.25 ± 4.44 fold level of TRPA1 expression (n = 4) relative to pooled controls compared to a 1.54 ± 0.58 fold expression in the individual control mice (n = 4) (p < 0.05, Control vs. EAE) (Fig. 3B). The pooled control is an equal part mixture of cDNA from each sample of the control group (i.e. from 4 animals). This mixture of control group cDNA was used as the reference sample for the purpose of the 2^−ΔΔCT^ method. Hydralazine-treated EAE mice, at 1 mg/kg daily starting from the day of induction (EAE + HZ, n = 5), exhibited a 6.32 ± 4.65 fold expression of TRPA1 relative to pooled controls, demonstrating a tendency to lessen the elevation of TRPA1 expression in EAE. However, these values in the EAE + HZ group were not significantly different from sham-treated EAE mice (p > 0.05).

### Hydralazine reduces acrolein adducts and myeloperoxidase in the EAE spinal cord

**Figure 4:**
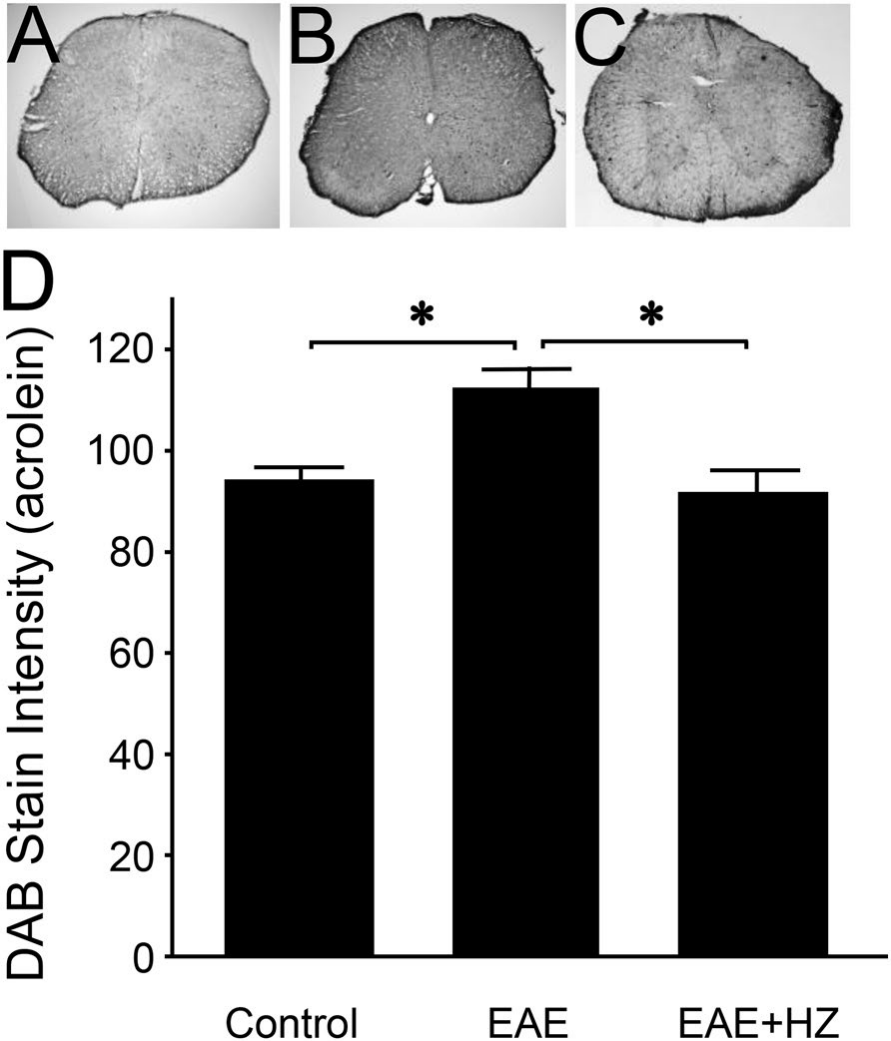
Immunostaining of FDP-Lysine in the spinal cords of EAE and hydralazine-treated mice. FDP-Lysine, an acrolein adduct, was significantly elevated in the spinal cord of EAE mice compared to controls on day 10 post induction. Treatment with hydralazine, a carbonyl scavenger, reduced the presence of FDP-Lysine compared to untreated EAE mice. No significant differences were observed in levels of FDP-Lysine between hydralazine treated animals and control. *p < 0.05.

**Figure 5:**
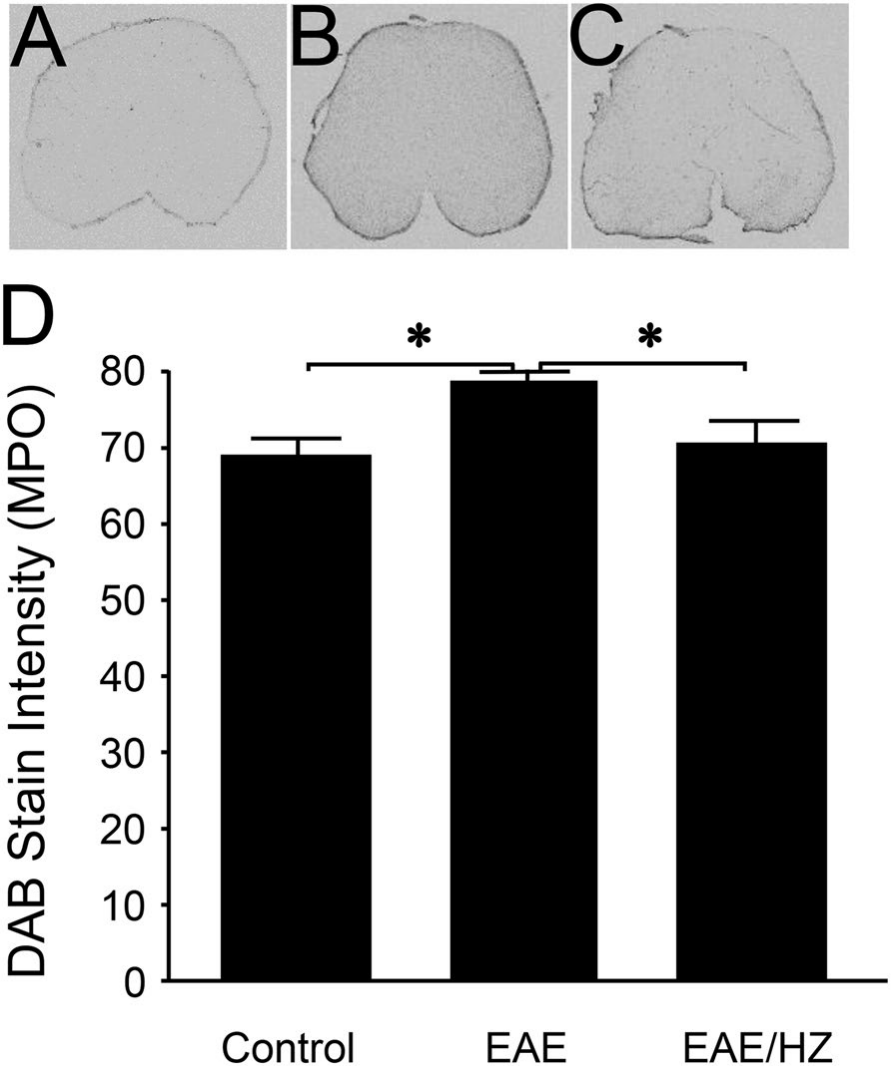
Immunostaining of myeloperoxidase in the spinal cords of EAE and hydralazine-treated mice. MPO was significantly elevated in the spinal cord of EAE mice compared to controls on day 10 post induction. Hydralazine treatment reduced the presence of MPO compared to untreated mice. No significant differences were observed between hydralazine treated mice and control animals. * p < 0.05.

Immunostaining revealed elevated levels of acrolein-modified lysine residues in the spinal cords of EAE mice (n = 5) compared to controls (n = 4) (p < 0.05) assessed in samples harvested on day 10 post induction (Fig. 4A,B,D). Tissue from hydralazine-treated EAE mice, at 1 mg/kg daily starting from the day of induction (EAE + HZ, n = 5), displayed significantly lower levels of acrolein adducts compared to the EAE group (p < 0.05) and were not significantly different than controls (p > 0.05) (Fig. 4B–D). Similarly, EAE mice (n = 5) exhibited higher levels of myeloperoxidase in the spinal cord compared to controls (n = 4) (p < 0.05), and these levels were reduced with hydralazine treatment, at 1 mg/kg daily starting from the day of induction (EAE + HZ, n = 4) (p < 0.05) (Fig. 5). Additionally, the levels of myeloperoxidase in the spinal cords of hydralazine-treated EAE mice were not significantly different than controls (p > 0.05) (Fig. 5A,C,D).

### Modulation of ALDH2 activity impacts acrolein levels in the spinal cords of EAE mice

**Figure 6:**
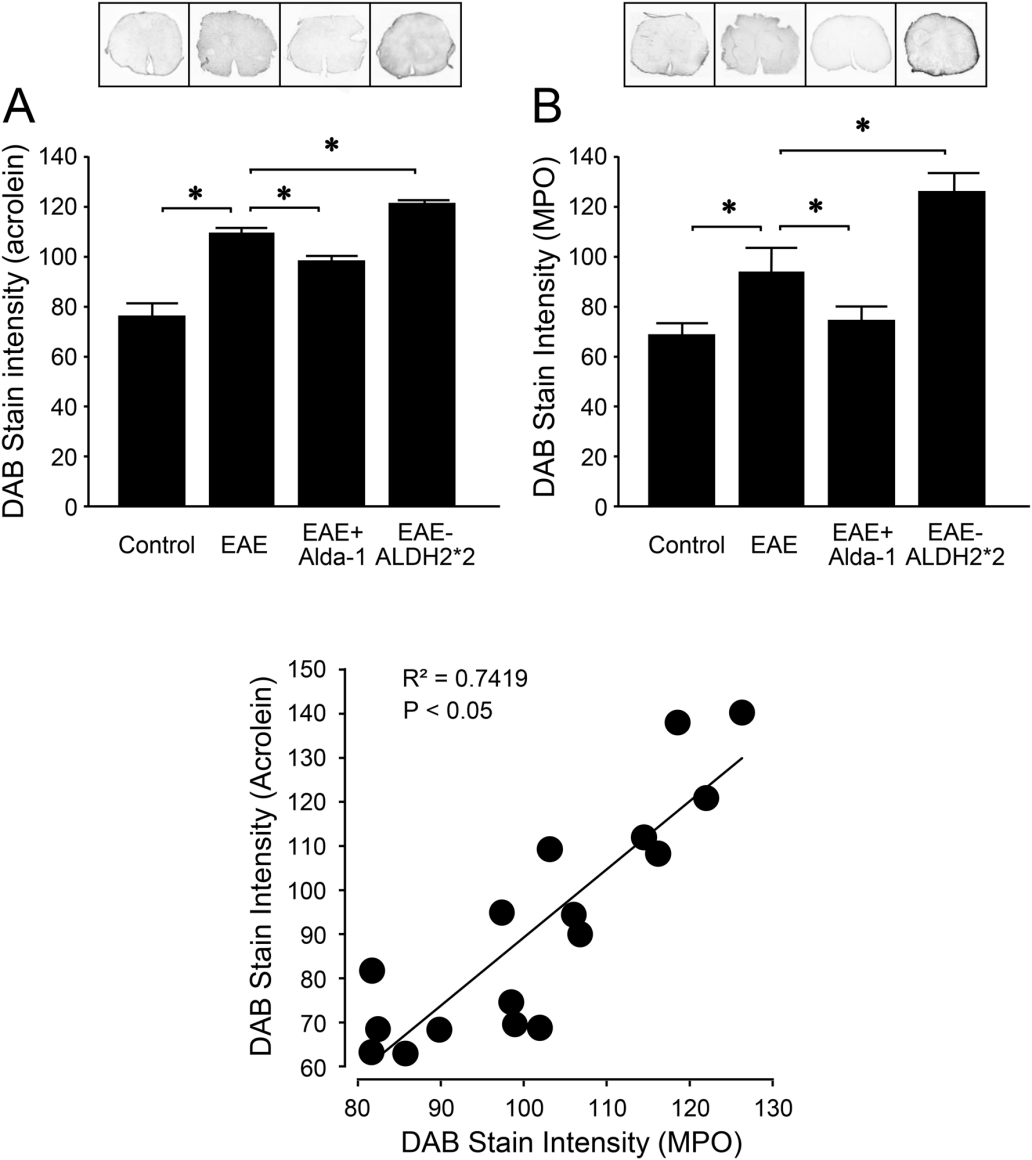
The effect of functional regulation of ALDH2 on the level of acrolein and MPO in spinal cord. (**A**) The comparison of the level of FDP-Lysine in the spinal cords of various conditions assessed on day 20 post induction. FDP-Lysine was significantly elevated in the spinal cord of wild-type EAE mice compared to controls. However, Alda-1 treatment in wild-type EAE mice suppressed levels of FDP-Lysine compared to untreated mice. Furthermore, ALDH2*2 mutant EAE mice exhibited increased FDP-lysine compared to wild-type EAE mice. No significant differences were observed between controls and Alda-1 treated wild-type mice. *p < 0.05. (**B**) The comparison of the level of myeloperoxidase in the spinal cord of various conditions assessed on day 20 post induction. MPO was significantly elevated in the spinal cord of wild-type EAE mice compared to controls. However, Alda-1 treatment in wild-type EAE mice suppressed levels of MPO compared to untreated mice. Furthermore, ALDH2*2 mutant EAE mice exhibited increased MPO compared to wild-type EAE mice. No significant differences were observed between controls and Alda-1-treated wild-type mice. *p < 0.05 compared to wild-type EAE. (**C**) Correlation of FDP-Lysine with myeloperoxidase. Linear regression of levels of FDP-Lysine and myeloperoxidase was performed to examine the relationship between the elevation of myeloperoxidase and acrolein generation. Regression analysis demonstrated a positive relationship with a significant correlation between levels of acrolein and myeloperoxidase (R^2^ = 0.7419, p < 0.05).

In this group of experiments, immunostaining once again revealed elevated levels of acrolein-modified lysine residues in the spinal cord of EAE mice (n = 5) relative to controls (n = 6) (p < 0.05), examined in samples harvested on day 20 post induction (Fig. 6A). Treatment with Alda-1 (50 mg/kg daily IP, started immediately after the induction), a known effective ALDH2 agonist, reduced the levels of acrolein-lysine adducts in the spinal cord of EAE mice (EAE + Alda-1, n = 6) when compared to EAE mice (p < 0.05). In contrast, ALDH2*2 mice, a type of transgenic mice with a known ALDH2 functional deficiency^58^, exhibited significantly elevated levels of acrolein adducts (EAE-ALDH2*2, n = 5) compared to wild-type EAE mice (p < 0.05) (Fig. 6A).

Similar to the observed elevation in acrolein adducts, immunostaining also revealed elevated levels of myeloperoxidase (MPO) in the spinal cords of EAE mice (n = 4) compared to controls (n = 4) (p < 0.05), analyzed in samples harvested on day 20 post induction (Fig. 6B). However, MPO levels were suppressed with Alda-1 treatment (50 mg/kg daily IP, started immediately after the induction, n = 5) and elevated in ALDH2*2 mice (n = 4) when compared to wild-type EAE mice (p < 0.05) (Fig. 6B). Linear regression of MPO and acrolein levels demonstrated a significant correlation between MPO and acrolein in all experimental conditions (R^2^ = 0.7419, p < 0.05) (Fig. 6C).

### Modulation of ALDH2 activity affects motor and sensory deficits in EAE Mice

**Figure 7:**
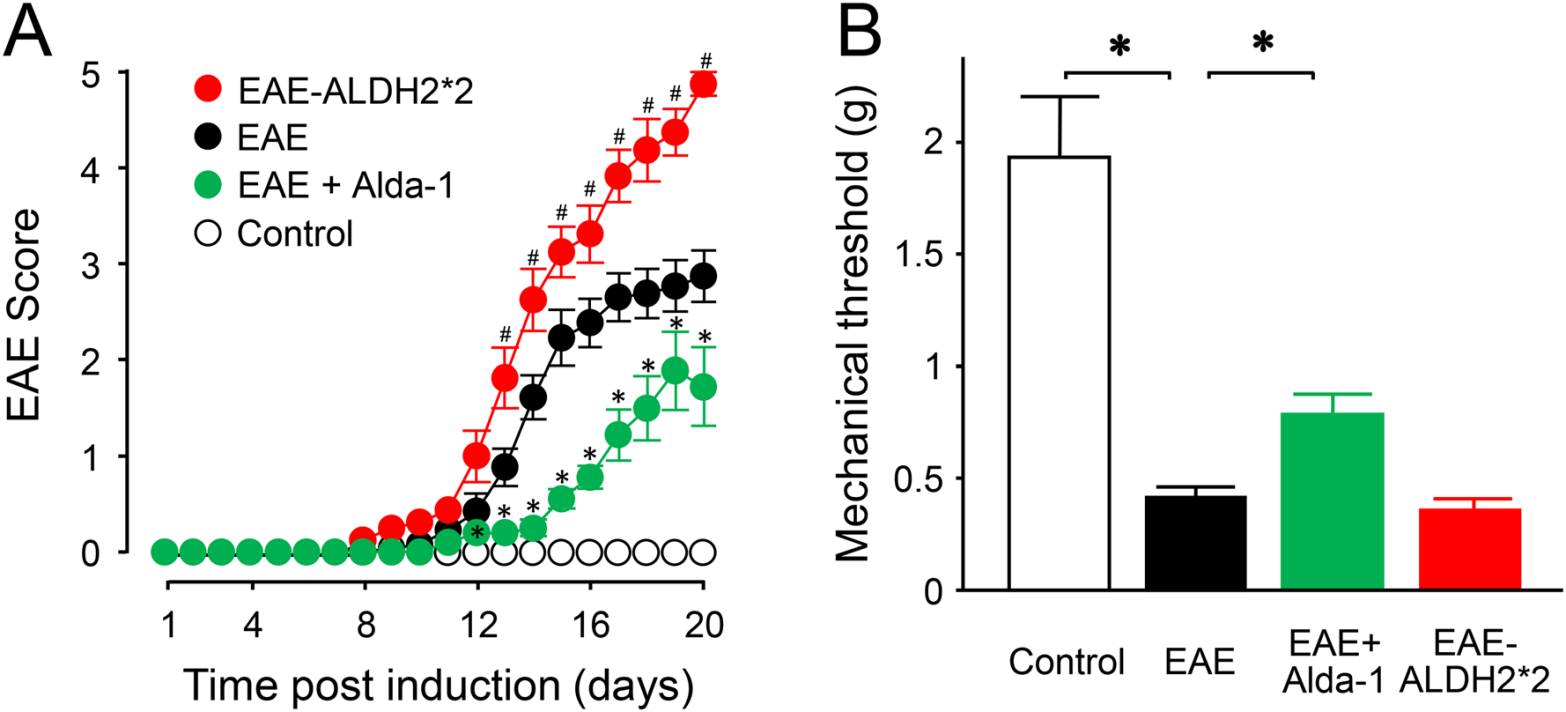
Modulation of ALDH2 activity resulted in correspondent changes in motor and sensory behavioral deficits. (**A**) Comparison of motor function of various conditions in wild-type and ALDH2*2 mice. While control mice displayed no functional deficits, wild-type EAE mice display typical motor deficits (black). The treatment of Alda-1, a drug which enhances ALDH2 activity, in wild-type EAE mice (green) commenced immediately after induction alleviated motor deficits on multiple days starting from day 12 post induction (*p < 0.05 compared to EAE). Conversely, ALDH2*2 knock-in mice (red), which have a deficient form of the enzyme, exhibited significantly more severe motor deficits when compared to wild-type EAE mice starting from day 13 post induction (*p < 0.05 compared to EAE). (**B**) Comparison of sensory hypersensitivity of various conditions in wild-type and ALDH2*2 mice. Wild-type EAE mice display significant hypersensitivity based on mechanical withdrawal thresholds at day 10 post induction compared to the control group. However, this hypersensitivity was significantly alleviated in Alda-1 treated animals, when commenced immediately post induction. While there was a tendency of worsening hypersensitivity in ALDH2*2 mice compared to wild-type EAE, no significant difference was observed between them. *p < 0.05.

In this group of experiments, we set out to determine whether up and down regulation of ALDH2 function in EAE mice using either pharmacological or genetic strategies could lead to correspondent motor and sensory changes. As shown in Fig. 7A, no motor deficits were observed in the wild-type control group (n = 10) during the experimental duration of 20 days post induction. However, typical motor deficits were recorded in the wild-type EAE group (n = 13). Treatment of this group with Alda-1 (50 mg/kg daily IP, started immediately after the induction, EAE + Alda-1, n = 10) significantly reduced the severity of motor symptoms starting from day 12 post induction when compared to wild-type EAE mice (p < 0.05). Conversely, ALDH2*2 mice (EAE-ALDH2*2, n = 8) exhibited significantly more severe motor symptoms than wild-type EAE mice starting at day 13 post induction (p < 0.05). Notably, four ALDH2*2 animals reached the humane endpoint for euthanasia from motor symptom severity between days 18 and 20. We continued to record the scores as 5 after euthanasia if the mice reached a humane endpoint. For the humane endpoints, the mouse either had to lose > 20% of body weight or reach a motor score of 5.

The effects of up and down regulation of ALDH2 activity on sensory hypersensitivity of EAE mice, assessed at 10 days post induction, is shown in Fig. 7B. Specifically, EAE mice displayed a reduced mechanical threshold (0.422 ± 0.042) (EAE, n = 13) when compared to controls (1.93 ± 0.27) (n = 10, p < 0.05). Treatment of the EAE group with Alda-1 (50 mg/kg daily IP, started immediately after the induction, EAE + Alda-1, n = 10) significantly mitigated this sensory hypersensitivity (0.79 ± 0.09) (p < 0.05). However, despite a tendency of augmenting the hypersensitivity, no significant difference of measurements using the von Frey filaments was observed between the ALDH2*2 (0.37 ± 0.063) (EAE-ALDH2*2, n = 8) and wild-type EAE mice (p > 0.05).

## Discussion

Although the precise etiology of multiple sclerosis is unknown, demyelination, inflammation, oxidative stress, and axonal degeneration are considered key components of its pathophysiology^1,3,5^. Previous works have identified acrolein, a pro-oxidant and pro-inflammatory aldehyde, as a potential mediator in the pathogenesis of experimental autoimmune encephalomyelitis (EAE)^19–24,27,28,31–33,59,60^. In this study, we have provided further evidence to strengthen the notion that acrolein is a key factor in EAE, not only as a pathological culprit, but also as a potential therapeutic target to deter the progression of neuronal degeneration in EAE. Specifically, in the current study, we have demonstrated that two known effective acrolein scavengers with distinct structures can alleviate motor deficits. In addition, hydralazine is shown to not only relieve motor but also sensory deficits when treatment started immediately post induction, further underscoring the neuroprotective role of acrolein sequestering.

We also investigated the effects of acrolein scavengers on myeloperoxidase (MPO), a proinflammatory enzyme known to catalyze the production of acrolein and that plays a key role in the pathogenesis of MS^42,43^. We have shown that acrolein sequestering induced by hydralazine in EAE mice was associated with a reduction of elevated levels of myeloperoxidase when examined at 10 days post induction, a period of symptom emergence (Figs. 4, 5). Furthermore, we observed that the levels of acrolein and MPO correlated significantly in various experimental conditions at 20 days post induction (Fig. 6), a timepoint that coincides with peak motor symptoms (Fig. 1)^19,22,61^. This finding is consistent with the established close interconnection between oxidative stress and inflammation, two key pathological culprits in MS^1,3–5,29,30^. While the anti-inflammatory and neuroprotective effects of hydralazine could be attributed to acrolein scavenging, other effects of hydralazine may also exist in this regard. For example, hydralazine has been shown to be capable of activating the neuronal Nrf2-ARE signaling pathway that is known to be neuroprotective^62–64^, a possibility in the context of hydralazine and EAE that remains to be examined.

In addition to exploring the neuroprotective effects of exogenously-applied acrolein scavengers, we investigated the role of an endogenous acrolein-scavenging machinery, the enzyme ALDH2^46^. We have found that up and down regulation of ALDH2, either through pharmacological treatment (enhancement by Alda-1), or genetic manipulation (suppression through altered genes), can lead to correlative changes of acrolein and MPO, indicating a key role of ALDH2 in influencing inflammation and oxidative stress in EAE. This is consistent with evidence that ALDH2 is a key regulator of aldehydes, while acrolein is known to promote inflammation^46,65–69^. In addition to biochemical changes, the functional alteration of ALDH2 was also associated with corresponding changes in motor dysfunction and sensory hypersensitivity (Fig. 7), suggesting a critical role of ALDH2 in the functional deficits of EAE. These findings not only further consolidate the key role of aldehydes in the pathology of EAE, but also elucidate the critical mechanisms that regulate their expression. This is expected to not only deepen our understanding of the acrolein-mediated destruction of myelin and neuronal loss, but further expand the possible therapeutic targets of anti-aldehyde pharmaceutical intervention to achieve neuroprotection through both endogenous and exogenous manners.

Currently, hydrazine compounds such as hydralazine are among the most well-studied acrolein scavengers^19,22,53–56,70–77^. In addition to hydralazine, the present study also examined phenelzine as an alternative scavenger to confirm whether the efficacy of hydralazine is due to hydrazine functionality or due to a mechanism specific to hydralazine through the phthalazine scaffold or other functional groups. Given that phenelzine, which is known to reduce acrolein in rodents^53,78^, produces a similar reduction of motor and sensory deficits when compared to hydralazine, it is likely that the effects of hydralazine and phenelzine are due to the hydrazine group, which readily forms a Schiff base with reactive carbonyls such as acrolein^72,79,80^. Taken together, we can conclude that the common feature of these two acrolein scavengers is their possession of functional groups capable of binding and neutralizing acrolein, which is likely the basis for their shared function of anti-acrolein-mediated neuroprotection.

The first use of acrolein scavengers to reduce acrolein expression in EAE was reported more than a decade ago by our group. In the initial treatment regimen, the acrolein scavenger was applied immediately following EAE induction, but before the emergence of symptoms^19^. A recent study administered an acrolein scavenger prior to the induction of EAE to achieve neuroprotection^23^. Our finding in this study constitutes the first report that commencing anti-acrolein treatment after the emergence of symptoms is still effective in reducing motor deficits (Fig. 1D,E). In our opinion, this finding makes this potential intervention more clinically relevant, as post-symptomatic treatment is more likely in the management of MS. Furthermore, the fact that delayed treatment is still effective suggests that the production of acrolein likely occurs throughout the disease progression and therefore can be mitigated through an extended use of pharmaceutical scavengers. Understandably, this widens the therapeutic window of effective anti-acrolein treatment for EAE beyond what has been shown in the current literature, with the possibility of its further expansion following future studies.

Neuropathic pain is a common symptom in patients with multiple sclerosis, with between 50 and 80% of patients experiencing some degree of pain^81–83^. This is thought to result from demyelination and neuroinflammation typical of the disease^38–40^. Thus far, efforts to treat neuropathic pain have had limited success in frontline therapies, providing relief in only 40–60% of cases^39^. Recent work in both a relapsing–remitting and chronic variant of EAE has also demonstrated that reactive carbonyls contribute to nociceptive behavior via the receptor channel TRPA1, and that TRPA1 antagonists can reduce mechanical allodynia in EAE mice^38,84,85^. Here, we found that acrolein scavenging treatments alleviated EAE-related nociceptive behaviors and reduced the presence of myeloperoxidase and acrolein adducts in the spinal cords of EAE mice. In addition, our quantification of TRPA1 mRNA concurs with previous findings of elevated TRPA1 transcripts in MOG-induced progressive EAE^85^. Interestingly, hydralazine-induced alleviation of neuropathic pain was not associated with a significant reduction of elevated TRPA1 mRNA. As such, it is likely that hydralazine reduced nociceptive behaviors associated with EAE primarily by scavenging acrolein. This thereby reduced the presence of TRPA1 agonists, but not TRPA1 expression directly, during our period of experimentation.

In this study, we have observed that EAE mice with the deficient ALDH2*2 mutation exhibited a more severe clinical progression and elevated acrolein adduct formation and myeloperoxidase compared to wild-type EAE mice. This indicates that the function of ALDH2 is important in suppressing acrolein levels in mice. Furthermore, dysfunction of ALDH2 can lead to more intensified oxidative stress and subsequently heightened pathologies in EAE. Interestingly, the ALDH2 polymorphism (ALDH2*2) is one of the most common genetic deficits in humans, found in over 560 million people worldwide^46^. Our study suggests that individuals with ALDH2 deficiency may have a higher risk of developing MS, in addition to experiencing more severe symptoms in relation to the EAE model compared to those without this genetic predisposition. This is expected due to the fact that ALDH2-deficient individuals have a compromised endogenous acrolein-sequestering system, which could lead to higher levels of acrolein accumulation. This thereby renders them more susceptible to MS development and a heightened progression of degeneration.

One relevant note about ALDH2 as a potential therapeutic target is the establishment of Alda-1, a catalytic stimulator of ALDH2 that enhances its enzymatic activity^65^. As shown in our study, Alda-1 can significantly reduce acrolein and MPO expression in addition to mitigating motor and sensory deficits in EAE. This is in good agreement with a previous work illustrating that increasing the activity of ALDH2 with Alda-1 treatment improves functional outcomes and decreases the presence of aldehyde metabolites in EAE mice^51^. Together, this and other studies have distinguished ALDH2 as a potential therapeutic target to detoxify acrolein. Furthermore, such strategies to galvanize the endogenous acrolein-sequestering system could be paired with exogenously-applied acrolein scavengers to further enhance acrolein removal and neuroprotection.

With a growing strength in evidence derived from preclinical animal studies, coupled with a recent case report of efficacy from a clinical observation, the prospect of anti-acrolein as a novel therapy for MS has been gaining momentum^37^. With an established, clear pathological mechanism and proven therapeutic target, coupled with the available clinical medications that have already shown initial success in lowering acrolein in humans, it is reasonable to speculate that anti-acrolein treatment is a novel potential therapy with significant translational power.

## Data Availability

The datasets used and/or analyzed during the current study are available from the corresponding author on reasonable request.
